# A Host of Factors Regulating Influenza Virus Replication

**DOI:** 10.3390/v2020566

**Published:** 2010-02-05

**Authors:** Andrew Mehle, Jennifer A. Doudna

**Affiliations:** 1 Department of Molecular and Cell Biology, University of California, Berkeley, CA 94705, USA; 2 Department of Chemistry, Howard Hughes Medical Institute, University of California, Berkeley, CA 94705, USA; 3 Physical Biosciences Division, Lawrence Berkeley National Lab, Berkeley, CA 94720, USA

## Abstract

A new series of genetic screens begins to illuminate the interaction between influenza virus and the infected cell.

Influenza virus is a brutally efficient pathogen; only eight viral genes encoding 11 major proteins direct all the processes needed for productive infection. But like other viruses, influenza virus exploits, and in some cases subverts, cellular proteins and pathways to promote replication. This phenomenon has long been appreciated for influenza virus, primarily at the level of discrete interactions between individual viral and host factors. Now, a recent collection of papers has begun to provide a global view of the interface between influenza virus and its host. Three independent research groups performed genome-wide siRNA silencing screens to characterize host proteins involved in influenza virus infection [1–3], complementing an existing screen performed in *Drosophila* (Table 1) [4]. An additional group utilized an integrative genomics strategy including results from yeast two-hybrid analysis, transcriptional profiling and siRNA silencing to identify viral-host interactions [5]. Results from these screens illuminate the complex relationship between influenza virus and the infected cell, and provide the foundation for a more comprehensive understanding of the viral life cycle (Figure 1).

Influenza virus relies on cellular processes throughout its replication cycle [6]. Briefly, influenza virus infection begins with attachment of virions to the cell by binding of the viral hemagglutinin (HA) protein to *N-* acetylneuraminic (sialic) acid moieties on the cell surface. The bound virus is endocytosed where, upon endosomal acidification, the HA protein mediates fusion between the viral envelope and endosomal membrane. Acidification also initiates the disassembly of the viral core and releases the genomic ribonucleoprotein (RNP) complexes into the cytoplasm. RNPs are imported into the nucleus where replication of the viral genome and transcription of viral genes occurs. Certain viral messages are spliced and all are exported to the cytoplasm for translation of viral proteins. New genomic RNPs are formed and exported to the cytoplasm where they assemble at the cytoplasmic side of the cell membrane with other viral proteins for budding and release of progeny virions.

The screens for factors regulating influenza virus replication identified host proteins required for steps throughout all stages of the infectious cycle. For example, vacuolar proton ATPases (vATPases) that acidify endosomes were abundantly represented in the four genome-wide siRNA screens [1–4]. ATP6AP1 and ATP6V0C were each identified in three separate screens. Individual knock-down of the vATPases strongly prevented viral influenza virus infection, confirming the initial hits [2–4]. vATPases have also been identified in RNAi-based screens as important host factors for infection by West Nile virus and Dengue virus, two other viruses that enter cells via a pH-dependent mechanism [7,8]. Detailed analysis confirmed that influenza virus entry was reduced in cells depleted of ATP6V0C and that an increased number of viral particles were retained in an early endosomal compartment [3]. By contrast, entry of viruses pseudotyped with the envelope from Moloney murine leukemia virus, which enters via pH-independent mechanisms, was unaffected by vATPase depletion. Complementing these findings, experiments using small molecules that inhibit vacuolar ATPases, bafilomycin A1 and concanamycin A from previous work and diphyllin from the current study, have been shown to reduce infection by blocking viral entry [3,9]. Thus, both chemical and genetic ablation of vATPase function disrupts influenza virus infection, reinforcing the critical role of endosome acidification for efficient viral entry and expanding the network of host proteins known to contribute to this process. The multiple RNAi-based screens unveiled cellular players in several other viral processes, including proteins involved in nuclear trafficking of viral factors (e.g. NXF1, KPNB1, XPO1, CSE1L/XPO2) and processing of viral mRNAs (e.g. CLK1).

While these screens identified factors involved in processes known to be important for viral infection, a significant advantage of such unbiased reverse genetic screens is the ability to detect interactions outside of those affecting known viral processes – they have the potential to identify new steps during viral replication and the host proteins that are involved. In the screens discussed here, such data implicate a variety of signaling pathways (WNT, NF-κB, p53, IP3-PKC, MAPK, PI3K-AKT and apoptosis), the ubiquitin-proteasome machinery, and the potential negative regulation of IFN-β production by viral polymerase proteins. Some of the most heavily represented genes that had not previously been associated with influenza virus infection are those encoding members of the COPI vesicle transport machinery. At least one variant of each of the subunits in the heptameric coatomer complex were detected as host factors important for virus infection. COPA, COPB1, COPB2, COPG, ARCN1, COPE and COPZ1 encoding the α-, β-, β′-, γ-, δ-, ε- and ζ1-COP proteins, respectively, were identified, often by multiple screens (Figure 1). The COPI complex functions in the early secretory pathway and is best characterized as mediating retrograde transport of luminal and membrane-bound proteins between the Golgi and the endoplasmic reticulum [10]. COPI proteins have also been implicated in endosomal transport and the pH-dependent entry of vesicular stomatitis virus and hepatitis C virus (HCV) [11–13]. The mode by which the COPI complex regulates influenza virus infection is unclear. A role of the COPI complex during viral entry is suggested by experiments showing that δ-COP (ARCN1) is required for fusion and deposition of viral proteins into the cytoplasm [3]. Conversely, depletion of β-COP (COPB1) reduced the levels of surface-exposed HA after infection, suggesting a role in its secretion [1]. It also remains possible that the COPI complex functions indirectly, whereby disrupted expression of the subunits alters the trafficking of other host factors that are important for viral replication or disrupts host membranes important for post-entry events, as has been suggested for HCV [11].

Combining results from all five screens permits further analysis of the host factors involved in influenza virus replication. A network created from all of the reported host factors yields a new MCODE cluster enriched for protein subunits of voltage-dependent calcium channels (Table 2).

Results from an earlier study of biologically active small molecules screening for their ability to perturb influenza virus replication identified several compounds that modulate Na^+^/Ca^2+^ exchange and the Ca^2+^-dependent protein kinase C [14], supporting a possible role of the related proteins in the MCODE cluster. In addition, König *et al.* identified the calcium sensor CAMK2B in their screen and propose that it plays a positive role in viral polymerase function [3]. Thus, Ca^2+^ regulation may be critical during influenza virus infection. A more in-depth analysis of all five studies is certain to identify additional regulators of infection.

Many of the potential host factors appear to function by facilitating, directly or indirectly, virus replication. Host factors that restrict virus replication were also detected. Perhaps the most compelling result from these screens was the identification by Brass, Huang and colleagues of the interferon-inducible transmembrane (IFITM) proteins that function as potent first-line antiviral defenders and antagonize viral infection [1]. IFITM3 was identified as an antiviral protein by showing that its knockdown significantly enhances viral infection. Little is known about the function of this conserved family of proteins, although knock-out studies show that they are not required for viability in mice [16]. Mouse embryo fibroblasts derived from a knockout strain lacking all IFITM genes (IFITM1, 2, 3, 5 and 6) were more susceptible to infection compared to their wild-type counterparts. Conversely, over-expression of IFITM1, 2 and 3 restricts viral replication. IFITM1 expression is induced in influenza infected cells [15] and IFITM2 and 3 were also identified as IFN-β regulated genes by Shapira *et al.* who showed that their silencing by siRNAs enhanced virus replication, but IFITM2 and 3 were not characterized in detail by these authors [5]. An elegant series of experiments by Brass *et al*. using psuedotyped particles showed that IFITM3 likely blocks infection early in the replication cycle during entry, representing a potentially novel antiviral process [1]. The exact mechanism by which entry is blocked, and whether this can be exploited to control influenza virus infections, remains to be determined. Expression of human IFITM3 in canine and chicken cells also prevented viral infection, suggesting that IFITM proteins utilize an evolutionarily conserved pathway to elicit an antiviral state. Yet, birds do not encode an obvious homologue to IFITM3. This raises the possibility that IFITMs might also influence viral tropism. Amazingly, IFITM1, 2 and 3 overexpression not only restricted virus-like particles bearing influenza virus HA proteins, but also blocked infection by particles coated with envelope proteins from three different flaviviruses: West Nile Virus, yellow fever virus, and Omsk hemorrhagic fever virus. IFITM3 was additionally shown to down-regulate replication of isolates of West Nile Virus and Dengue Virus. Thus, the IFITM proteins are a new class of antiviral restriction factors that disrupt infection of diverse viruses during early stages of replication.

These screens have provided an abundance, maybe even an overabundance, of likely influenza virus host factors. Out of 1539 total hits obtained in the five screens discussed here, 1417 are unique. Thus, only ∼8% of identified genes is common to at least two screens. No gene was shared by all 5 screens. Why? A similar pattern emerged from comparisons between multiple screens of host factors for HIV and several explanations have been proposed [17,18], most of which apply here. In particular, each study utilized different experimental approaches. For example, unlike the genome-wide siRNA screens performed in human cells, the screen conducted by Hao *et al.* was performed in *Drosophila* cells and Shapira *et al.* utilized an integrative genomics approach (Table 1) [4,5]. And while the integrative approach combined results from yeast two-hybrid, expression profiling, IFN-β production and viral replication assays, these results require additional validation. The protein:protein interactions suggested by two-hybrid analysis need to be confirmed. Furthermore, differences in mRNA levels during infection as detected by microarrays may not solely be a transcriptional response, but rather result from the destruction of host messages during synthesis of viral mRNA, disruption of cellular pre-mRNA processing, or preferential export of viral mRNA from the nucleus [19]. Such differences in screening, infection assays and data analysis almost certainly limit the type of factors that can be identified and the coincidence of factors between screens. The possibility that the screens might also possess high false positive and false negative rates which would contribute to the large number of genes identified and low overlap between data sets cannot be eliminated.

If just the genome-wide screens in human cells are considered, 839 genes were identified with only ∼5.8% overlap between at least two screens, and only four genes were common hits in these screens (Figure 1) [1–3]. The shared genes include the vATPase ATP6AP1 and COPI vesicle transport proteins ARCN1, γ-COP and β′-COP, suggesting crucial roles for these proteins in influenza virus replication. Yet even in these screens, the difference between single- and multi-cycle infection assays likely influenced the host partners that can be detected; single cycle infection assays, while simplifying the technical aspects of the screens, have the potential to only detect factors required for early steps of viral infection. The use of different cell types in these screens also raises the possibility that influenza virus might use distinct host proteins depending on cell type. If so, by extension, might influenza virus utilize different host factors depending on whether the host is a human, a bird, or a pig? And might this affect species tropism and zoonotic transmission? Despite the low overlap, bioinformatic and network analyses strengthen the combined results. The fact that members of the same multi-component macromolecular assemblies or pathways were repeatedly identified across the different experimental platforms provides added confidence that these genes encode *bona fide* host factors for influenza virus. The truly exciting results presented in these screens set the stage for future experiments to determine the detailed molecular mechanisms by which host proteins affect all aspects of viral biology. Such studies will be vitally important for understanding the interface between the virus and host.

## Figures and Tables

**Figure 1.**
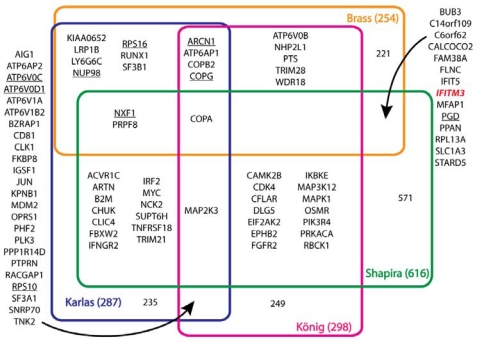
Identification of host proteins that regulate influenza virus replication in multiple screens. The overlap between different screens for influenza virus host factors is shown as a Venn diagram. Total genes for each screen and the number of genes unique to each screen are indicated. Note that each screen utilized different criteria to query and score host genes as regulators of virus infection. Genes found by Hao *et al.* [4] with obvious human homologues are underlined.

**Table 1.** Comparison of screens performed to identify host factors for influenza virus infection.

Authors	Virus	Target Cell	Assay(s)	Total hits	Replication?	Notes
**Brass *et al.* [1]**	A/PR/8/34	U2OS	immunostaining	254	single-cycle	Identified IFITM proteins as potent antiviral restriction factors
**Hao *et al.* [4]**	Flu-VSV-G-*Renilla* luciferase	DL1	luciferase assay	∼84 with obvious human homologues	single-cycle	Initial screens were performed in *Drosophila* cells using VSV-G psuedotyped reporter virus. Select hits were confirmed in human 293 cells with A/WSN/33 and an H5N1 isolate (A/Indonesia/7/2005).
**Karlas *et al*. [2]**	A/WSN/33	A549	immunostaining to measure primary infection, luciferase assay to titer virus yields	287	single- and multi-cycle	Many hits were confirmed using a 2009 H1N1 isolate (A/Hamburg/04/2009) and a H5N1 isolate (A/Vietnam/1204/2004).
**König *et al.* [3]**	A/WSN/33-Ren	A549	live-cell luciferase assay at 12, 24, and 36 h post-infection	298	single-cycle	HA coding sequence in the virus was replaced with the *Renilla* luciferase reporter gene. Many hits were confirmed with a 2009 H1N1 isolate (A/Netherland/602/2009).
**Shapira *et al.* [5]**	A/PR/8/34 (WT and ΔNS1)	HBEC	luciferase assay to titer virus yields	616	multi-cycle	Screening included analysis of yeast 2-hybrid, transcriptional profiling, IFN-β production and infection assays.

**Table 2.** Voltage-dependent calcium channel MCODE cluster.

CACNA1A [5]	CACNG1 [5]
CACNA1G [5]	CACNG4 [3]
CACNB1 [5]	CACNG7 [5]
CACNB3 [5]	RASGRP2 [5]
CACNB4 [1]	

References are as assigned in text.
